# The lncRNA epigenetics: The significance of m6A and m5C lncRNA modifications in cancer

**DOI:** 10.3389/fonc.2023.1063636

**Published:** 2023-03-09

**Authors:** Vincenza Ylenia Cusenza, Annalisa Tameni, Antonino Neri, Raffaele Frazzi

**Affiliations:** ^1^ Laboratory of Translational Research, Azienda Unità Sanitaria Locale - IRCCS di Reggio Emilia, Reggio Emilia, Italy; ^2^ Scientific Directorate, Azienda Unità Sanitaria Locale - IRCCS di Reggio Emilia, Reggio Emilia, Italy

**Keywords:** lncRNA, m5C, epigenetics, cancer, m6A

## Abstract

Most of our transcribed RNAs are represented by non-coding sequences. Long non-coding RNAs (lncRNAs) are transcripts with no or very limited protein coding ability and a length >200nt. They can be epigenetically modified. N6-methyladenosine (m6A), N1-methyladenosine (m1A), 5-methylcytosine (m5C), 7-methylguanosine (m7G) and 2’-O-methylation (Nm) are some of the lncRNAs epigenetic modifications. The epigenetic modifications of RNA are controlled by three classes of enzymes, each playing a role in a specific phase of the modification. These enzymes are defined as “writers”, “readers” and “erasers”. m6A and m5C are the most studied epigenetic modifications in RNA. These modifications alter the structure and properties, thus modulating the functions and interactions of lncRNAs. The aberrant expression of several lncRNAs is linked to the development of a variety of cancers and the epigenetic signatures of m6A- or m5C-related lncRNAs are increasingly recognized as potential biomarkers of prognosis, predictors of disease stage and overall survival. In the present manuscript, the most up to date literature is reviewed with the focus on m6A and m5C modifications of lncRNAs and their significance in cancer.

## 1 Introduction

The majority of our transcribed RNA are non-coding sequences, since just 2% of all the transcribed RNA is translated into polypeptides (1). Long non-coding RNAs (lncRNAs) are transcripts with no or very limited protein coding ability and a length >200nt. Within their sequence incorporate multiple interaction sites, responsible for the recruitment of the molecular partners. These interactions contribute to the specific localization and functions of lncRNAs (2).

The RNA can be epigenetically modified and RNA modifications are highly dynamic, some of them reversible, and represent a pivotal component of the post-transcriptional gene regulatory landscape (3).

Several epigenetic modifications involving lncRNAs are relevant in human cancer. Namely, these are N6-methyladenosine (m6A), N1-methyladenosine (m1A), 5-methylcytosine (m5C), 7-methylguanosine (m7G) and 2’-O-methylation (Nm) (4).

The focus of this review is the epigenetic modification of lncRNAs, a class of molecules actively involved in carcinogenesis and tumor progression of both common (like colorectal cancer) and rare (like uveal melanoma) tumors (5, 6). A variety of lncRNAs, like metastasis associated lung adenocarcinoma transcript 1 (*MALAT1*), the Hox transcript antisense intergenic RNA (*HOTAIR*), or X inactive specific transcript (Xist) have been linked to cancer in recent years (7–9). *MALAT1*, when expressed, promotes the proliferation of tumor cells. Indeed, *MALAT1* has been found highly expressed in different types of solid and haematological malignancies. This lncRNA exerts its tumorigenic role by regulating several functions like mRNA splicing and transcription, gene and miRNA expression, activation of miRNAs targets (7). *HOTAIR* is aberrantly expressed in solid tumors, where maintains proliferation through the evasion from the growth inhibitors, the induction of vasculogenesis, the activation of invasion and metastasis, the genomic instability (8). *Xist* is dysregulated in various malignancies. Elevated *Xist* expression associates with poor prognosis and disease-free survival, larger tumor size, metastasis and tumor stage (10, 11).

Three classes of enzymes control the epigenetic modification of RNA: “writers”, “readers” and “erasers”. Each class play a role in a specific phase of the modification and m6A and m5C are the most represented modifications in RNA of mammalian cells (4). The writers add a methyl group to the specific nucleotides (adenines or cytosines); the readers recognize the modified adenines or cytosines and exert a specific function while the erasers have the role of removing the markers (12).

The m6A consists in the addition of a methyl group to the adenine in position 6 by the m6A “writer” complex (13). This latter is a highly conserved mRNA methyltransferase complex, including various m6A methyl group transferring proteins (METTL3, METTL14, WTAP, KIAA1429, RBM15, RBM15B, METTL16) (12). In mRNA, it constitutes approximately 0.1 – 0.4% of the total amount of adenines (14). m6A “writer” complex is also responsible for the methylation of the adenine in position 6 of coding and non-coding RNAs. m6A-modified site recognizing proteins (YTHDF1, YTHDF2, YTHDF3, YTHDC1, YTHDC2, elF3, HNRNPA2B1, HNRNPC), and methyl group removing proteins (ALKBH5, FTO) are the other two classes of enzymes responsible for the function or the remodelling of this epigenetic change (12).

Primarily considered a DNA modification, m5C is also present in the RNA. It locates downstream of the translation initiation site and in the UTRs of mRNA but also in rRNA and lncRNA (15). The currently known RNA m5C-methyltransferases are more than eight. These belong to two families of methyltransferases: the NOL1/NOP2/SUN domain (NSUN) family that contains several variants (NSUN1 to NSUN7) and DNA Methyltransferase homolog 2 (DNMT2) family, initially considered a DNA methyltransferase (15). Two are the m5C readers identified: the Aly/REF export factor (ALYREF) and the Y-box-binding protein 1 (YBX1) (16). Little is known about the m5C “erasers” though. There is some evidence that this role could be played by the ten-eleven translocation enzymes (TETs), already known to catalyze the reaction of DNA hydroxymethylation (5hmC) (17).

The epigenetic-modified lncRNAs may also be structured in signatures that demonstrated to have a prognostic role in tumors (18).

Overall, the great interest raised by the epigenetics of lncRNA in human tumors deserves further and deeper understanding with the aim of identifying those modifications affecting structure and functions of lncRNAs.

## 2 m6A modification

**Figure 1:**
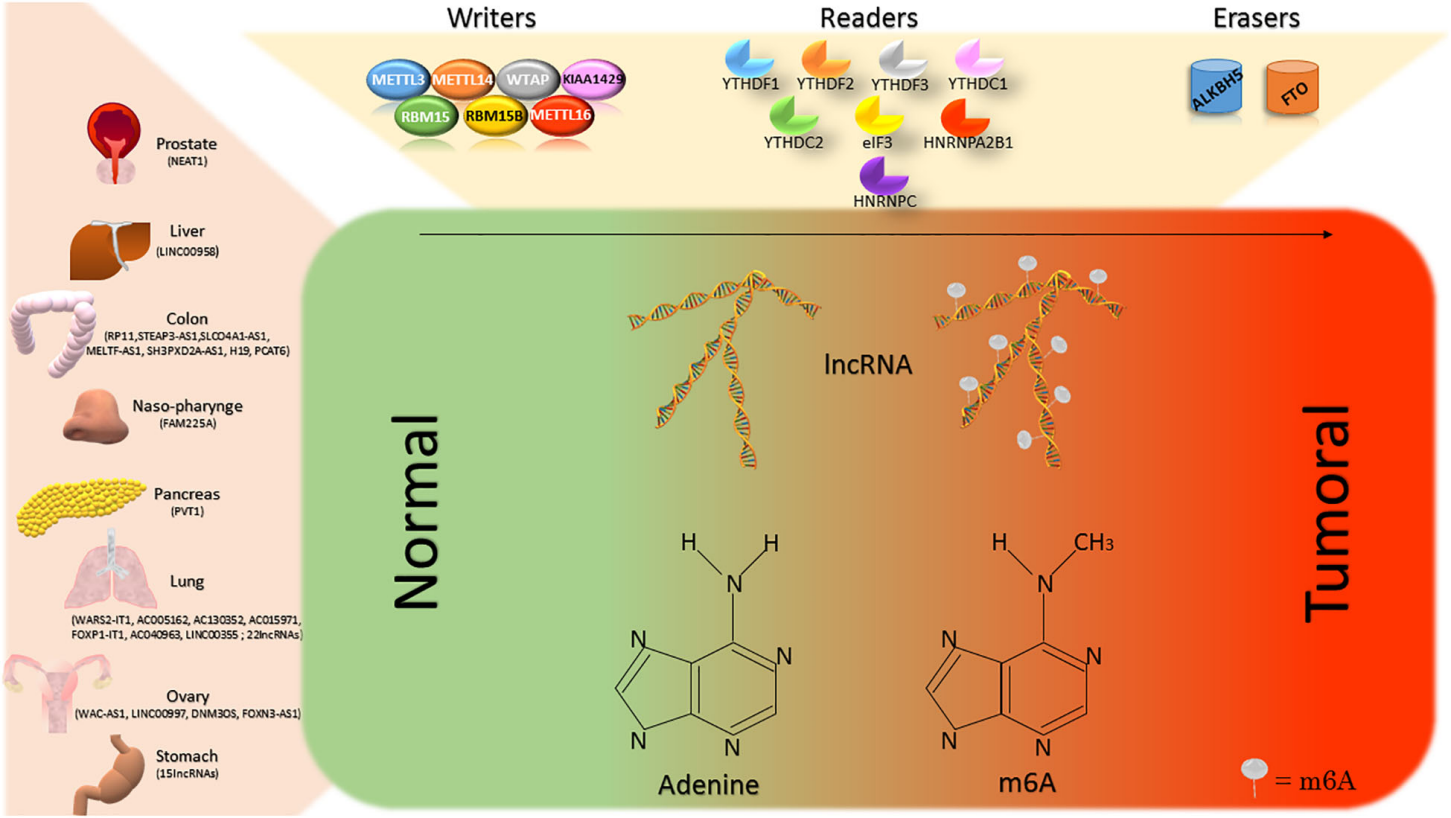
The lncRNAs of different organs are subject to the action of m6A-modifying enzymes. The writers, readers and erasers contribute to the balance of the m6A levels on lncRNAs. In most of the cases, increasing m6A on lncRNAs determines a switch from normal to tumor tissues.

Post-transcriptional modifications are chemical changes that alter structure and properties, thus modulating the functions and interactions of lncRNAs. The epigenetic modification of RNA, carried on through the concerted action of the already mentioned classes of enzymes, modulates the turnover, stability and function of target RNAs (**Figure 1**) (4).

The m6A modification is established by a macromolecular protein complex, constituted by the core METTL3 and METTL14 and by their cofactors WTAP, VIRMA, ZC3H13, CBLL1, RBM15/15B. Another writer is the recently discovered METTL16. METTL3 belongs to the class I methyltransferase family and its depletion directly decreases m6A levels in the nucleus and cytoplasm. m6A establishment by m6A methyltransferase complex modulates lncRNAs function or expression (12).

A large amount of evidence links m6A epigenetic modification of lncRNAs to cancer prognosis and therapy response. For example, high levels of m6A *NEAT1* correlate with bone metastasis and the overexpression of this lncRNA induce cancer cell metastasis in mouse model through an m6A-dependent mechanism. *NEAT1* is one of the main cancer-related lncRNAs, overexpressed in several types of tumors and correlated to a worse patients survival (19). It contains four m6A sites, distributed along the whole region from 5’-3’ and performing specific functions. For instance, m6A site number 4 is responsible for the binding of *NEAT1* to CYCLINL1 (20). Since CYCLINL1 is a bone-specific protein, highly expressed in bone metastasis of prostate cancer, this is consistent with the role played by m6A-*NEAT1*. Furthermore, RNA-seq experiments unveil that m6A-*NEAT1* recruit CYCLINL1 and CDK19 on RUNX2 promoter through a RNA-DNA interaction. RUNX2 is an established driver of bone metastasis prostate cancer, thus strengthening the central role of m6A-*NEAT1* in prostate cancer bone metastasis (20).

m6A modifications add complexity and diversity to lncRNA modulatory potential. In particular, METTL3 upregulates *LINC00958* lncRNA increasing its stability and promoting hepatocellular cancer progression. METTL3 also increases *FAM225A* lncRNA stability in nasopharyngeal carcinoma and upregulates *RP11* lncRNA by increasing its nuclear accumulation in colorectal cancer (21). Furthermore, m6A regulates the relationship between lncRNAs and specific DNA sites through the formation of RNA-DNA triple helix structures. On the other side, m6A modification provides the binding sites for RNA readers or modifiers, such as RNA-binding proteins (RBPs). m6A readers are a class of binding proteins able to decode the m6A marks and affect the stability of methylated RNA (22).

Specific and direct role of lncRNAs and m6A modifying complexes associate with several human cancers (12, 23). The most accepted hypothesis for the role of m6A, is that m6A modification functions by altering the RNA structure or recruiting m6A readers like RBP (YTHDF1/2/3), YTH domain-containing 1/2 (YTHDC1/2), insulin-like growth factor 2 mRNA binding proteins 1/2/3 (IGF2BP1/2/3), heterogeneous nuclear ribonucleoproteins (HNRNPs) and zinc-finger CCCH domain-containing protein 13 (ZC3H13) (21). The demethylation of m6A sites is catalyzed by erasers, mainly represented by FTO and ALKBH5 ensuring that the epigenetic modification is reversible and that there is an equilibrium of m6A in the transcriptome (24). The activity of the eraser ALKBH5 determines m6A demethylation on both single-stranded RNA and DNA. ALKBH5 may act as tumor suppressor in pancreatic cancer but on the contrary, as tumor promoter in osteosarcoma *via* upregulation of *PVT1* lncRNA (21).

m6A-related lncRNAs (mRLs) signature have been demonstrated to have prognostic value and can predict the overall survival of patients affected by cancer (25–30). A more detailed description will follow below in this manuscript.

The hypoxia condition represents a tumor-promoting factor for many solid tumors. It has been recently demonstrated by Zhou Li et al. and colleagues that hypoxia-inducible factor-1α (HIF-1α) controls the tumor progression of colorectal cancer *via* the stabilization of mRLs (31). Hypoxia drives the increase of HIF-1α, a transcription factor controlling several programs required for cancer cell malignancy like proliferation, stemness, metabolism, drug resistance and metastasis (32). The lncRNAs known to promote tumor progression under hypoxic conditions encompass *NEAT1, MALAT1, MIR31HG, RAB11B-AS1* and the recently discovered *STEAP3-AS1* (31). Through a Methylated RNA Immunoprecipitation (MeRIP) approach, the authors show that *STEAP3-AS1* lncRNA affects STEAP3 mRNA stability by binding to the YTHDF2 reader, thus preventing m6A mediated degradation of STEAP3 mRNA. Ultimately, this leads to an activation of the Wnt/β-catenin signaling pathway and to the progression of colorectal cancer (31).

## 3 m5C modification

**Figure 2:**
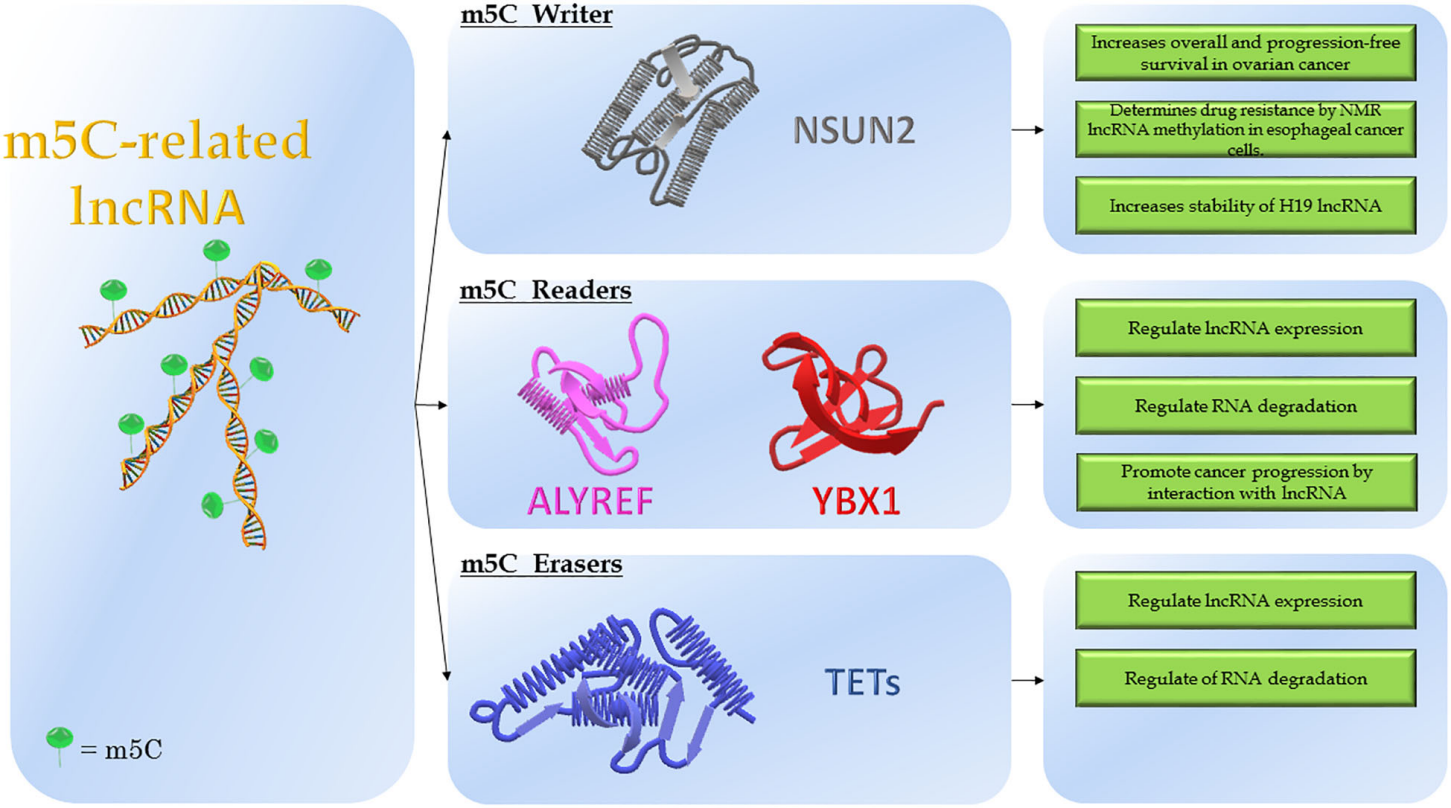
Modulation of m5C-related lncRNAs. The figure shows the molecular structure of lncRNA and m5C-modifying enzymes. On the left is reported the lncRNA modified by adding the m5C marker, named: m5C-related lncRNA. In the middle, the three classes of modifying enzymes: NSUN2 (“Writer”); ALYREF and YBX1 (“Readers”); TETs (“Erasers”). On the right, the molecular consequences depending on the effects of modifications.

Highly conserved in different species and distributed in a species-specific manner in various RNA types, the m5C functions remain unclear (16, 33). The biological effects exerted by m5C are mainly the regulation of RNA localization, stability and transcription efficiency (16). Similarly to the establishment of m6A methylation, the enzymatic systems encompass methyltransferases (“Writers”) that use the S-adenosylmethionine (SAM) as the methyl group donor, m5C binding proteins (“Readers”) that promote a specific function and demethylases (“Erasers”) that remove the m5C marker (**Figure 2**) (16, 34).

To date, NSUN2 is the only one that seems to perform methyltransferase function in lncRNAs, along with mRNA and tRNA (15, 16, 35). In esophageal cancer cells, NSUN2 binds and methylates *NMR* (nucleotide metabolism regulator) lncRNA, whose expression associates with esophageal cancer resistance to cisplatin or paclitaxel (36). In hepatocellular carcinoma, NSUN2 methylates and increases the stability of the tumor-related lncRNA *H19*. m5C-methylated *H19* correlates to poor hepatocellular carcinoma differentiation. Many cancers show abnormal *H19* overexpression and tumorigenic effects (37).

Cellular proliferation is another process where NSUN2 plays a role, affecting the expression and translation of key cell cycle regulators. Furthermore, it regulates the cellular senescence acting as a stress sensor. Taken together, the functional roles of NSUN2 suggest that m5C has important cellular roles (38). NSUN2 associates also with tumorigenesis and cell migration in colon cancer, gallbladder and bladder carcinoma. Additionally, it correlates with metastasis progression in breast cancer and gastric cancer after the SUMOylation by SUMO-2/3 (16, 38–42).

As far as today, two m5C readers have been identified: the Aly/REF export factor (ALYREF) and the Y-box-binding protein 1 (YBX1) (16).

In several cancers, ALYREF correlates to patients’ survival. Breast cancer displays high ALYREF expression that correlates to poor survival. In breast cancer cells, ALYREF influences cellular growth, apoptosis and mitochondrial energy metabolism through lncRNA *NEAT1*. Clecand co-authors, demonstrated that the short isoform of the lncRNA *NEAT1* is a molecular trigger for ALYREF effects in breast cancer. ALYREF regulates the expression of the short *NEAT1* isoform by binding directly *NEAT1* and stabilizing CPSF6. This latter is a protein implicated in the selective activation of the post-transcriptional generation of the short isoform of *NEAT1* (43). ALYREF regulates also the degradation of the unwanted mRNA and lncRNA by nuclear exosome, demonstrated in HeLa and HEK293 cells. Exosome‐mediated degradation uses the cofactor hMTR4. This latter recruits the exosome to its targets. Fan J. and co-workers showed that hMTR4 competes with ALYREF to bind ARS2. The competition reported above is necessary to determine the fate of an RNA. When ARS2 interacts with ALYREF, it recruits this latter to the RNA. If RNP factors stabilize the interaction, the RNA goes into the cytoplasm. On the contrary, if ALYREF cannot interact with ARS2 or RNP factors, hMTR4 then links ARS2 on an RNA and recruits the exosome (44).

YBX1 is a DNA- and RNA-binding protein involved in various processes. Translational repression, RNA stabilization, mRNA splicing, DNA repair and transcription regulation are some of the processes exerted by YBX1.

Literature reports that YBX1 interacts with different lncRNAs and mediates some key processes in cancer. In lung adenocarcinoma, YBX1 binds *LINC00472*, mediates changing in biophysical properties of cells and inhibits the migration and invasion of the carcinoma. This is mediated by a decreasing amount of free YBX1 that activates the translation of Snail mRNA. Thus, the low levels of YBX1 determine a decrease of Snail expression and inhibit the EMT of the cells (45). In 2020, Zhang et al. and co-workers demonstrated that the interaction between *DSCAM-AS1* and YBX1 promotes cancer progression through the activation of FOXA1 transcription network in lung adenocarcinoma, breast and prostate (46).

The 5mC-demethylase function can be carried on by the dioxygenases. TET family and ALKBH1 are reported to have a role in 5mC demethylation. ALKBH1 was reported, in 2022, to determine minor modification sites also in lncRNAs. However, it was reported that the main function of ALKBH1 is exerted on tRNAs (47).

It was reported in recent years that TET enzymes are also able to demethylate the RNA, in addition to DNA (48). One of the first demonstrations of the TET’s ability to hydroxymethylate the m5C in RNA is described in the 2014’s work by Fu and co-workers (17). The authors reported that the overexpression of TETs increase the hm5C levels in HEK293T cells. The relative levels of hm5C are lower in RNA than DNA, with a frequency of one hm5C every 5000 m5C. All the three TET enzymes could serve to hydroxymethylate the RNA but the authors speculate that the principal enzyme is TET3. This hypothesis is supported by the fact that, while TET3 localize in both nucleus and cytoplasm, TET1 and TET2 are exclusively in the nucleus. They hypothesize that TET enzymes contribute to the RNA demethylation, but TETs are not exclusive for the oxidation of m5C into hm5C and other actors could be involved (17). Furthermore, it has been reported that TET2 has a tolerance for m5C but prefers 5mC for conformational and steric reasons (49). The presence of hm5C RNA is functional in biological processes. It’s currently assumed that hm5C may represent the signal for RNA degradation and, in accordance to this hypothesis, colorectal and hepatocellular carcinomas miss hm5C RNA (50). TET2 also regulates the lncRNA *ANRIL* by binding to its promoter in gastric cancer. In addition, it controls the *ANRIL*-downstream genes. The *ANRIL* knockdown impairs the effect of TET2 on the proliferation and colony formation in gastric cancer. The TET2 mRNA levels correlate with the stage of the tumor, decreasing with high cancer stage. Since *ANRIL* is upregulated in gastric cancer and it is associated with a poor prognosis, it inversely correlates with TET2 mRNA levels (51, 52). In Acute Myeloid Leukemia, TET2 can activate lncRNA *MEG3* transcription. TET2 mediates the *MEG3* first intron DNA methylation. The presence of putative regulatory elements in the first intron of *MEG3* and *MEG3* expression may be repressed by hypermethylation as a result of lack of functional TET2, contributing to *MEG3* epigenetic silencing that occur specifically in the TET2-mutant AML subtype (53). Lyu et al. and co-workers proved that to increase *MEG3* expression, TET2 acts as a cofactor of WT1. WT1 is a transcriptional regulator that is capable of activating or repressing gene transcription (54).

During constitutive hypoxic conditions, TET’s hypomethylation in myeloid leukemia cells on WT1-intron1 CpG Island determines the transcription of its antisense-oriented lncRNA. The expression of the lncRNA is necessary for WT1 mRNA expression that seem to mediate the cell quiescence (55).

## 4 m6A-related lncRNAs signatures

Recent studies pointed out the importance of post-transcriptional modification in regulating lncRNAs networks and circuitries. The differential methylation status and specific methylation patterns of lncRNAs in particular play pivotal functions. To date, mRLs signatures are increasingly recognized as potential biomarkers of prognosis, predictors of disease stage and overall survival in several malignancies. Indeed, the characterization of mRLs is favoured by the possibility to easily detect their levels in body extracellular fluids (e.g., blood, urine), providing a milestone for the development of new prognostic/diagnostic tools and therapies (25). The manuscript published so far start from a TCGA data mining followed by bioinformatics and statistical analysis. The lncRNAs signatures can be validated in each specific model and the list of tumors described in the last three years is already consistent and reported in **Table 1**.

**Table 1:** m6A-related lncRNAs signatures.

Malignancies	m6A-related lncRNA signature	References
Breast cancer (TCGA-BRCA) (Adenomas and Adenocarcinomas, Adnexal and Skin Appendage Neoplasms, Basal Cell Neoplasms, Complex Epithelial Neoplasms, Cystic, Mucinous and Serous Neoplasms, Ductal and Lobular Neoplasms, Epithelial Neoplasms, NOS, Fibroepithelial Neoplasms, Squamous Cell Neoplasms)	AL359076.1, MFF-DT, AC114947.2, MIATNB, FARP1-AS1, AC106028.2, U73166.1, AP000919.3, ZNF197-AS1, AP005131.2, SP2-AS1,AL592301.1, OTUD6B-AS1, AL138789.1, COL4A2-AS1, AC024145.1, AL513218.1, LRRC8C-DT, AL021707.4, MIR302CHG, AC012442.2	(56)
Breast cancer (TCGA-BRCA)	Z68871.1, AL122010.1, OTUD6B-AS1, AC090948.3, AL138724.1, EGOT	(57)
Osteosarcoma (TARGET-OS) (osseous and chondromatous neoplasms)	AP003119.2, LINC01816, AL139289.1, AC004812.2, AC005785.1, AL353804.1	(58)
Gastric cancer (TCGA-STAD) (Adenomas and Adenocarcinomas, Cystic, Mucinous and Serous Neoplasms)	AL049840.3, AC008770.3, AL355312.3, AC108693.2, BACE1-AS, AP001528.1, AP001033.2, AC092574.1, AC010719.1, AC009090.3, SAMD12-AS1	(59)
Glioma (TCGA-GBM) and (TCGA-LGG)	AL390755.1, AL445524.1, AL359643.3, LINC00641, AL117332.1, LNCTAM34A, CRNDE, AP001486.2, CARD8. AS1	(60)
Pancreatic ductal adenocarcinoma (TCGA-PDAC)	AP005233.2, AC092171.3, AC010175.1, CASC8, TP53TG1, SNAI3.AS1, FLRT1, AC022098.1, DCST1.AS1	(61)
Clear-Cell Renal Cell Carcinoma (ccRCC) (TCGA-KIRC)	AC009948.2, AC011752.1, AC018752.1, AF117829.1, AL008718.3, AL133243.3, AL158071.5, COL18A1-AS1, DLEU2, LINC00115, RPL34-AS1, SNHG10	(62)
Lung Squamous Cell Carcinoma (TCGA-LUSC)	AL122125.1, HORMAD2-AS1	(63)
Lung Adenocarcinoma (TCGA-LUAD) (Acinar Cell Neoplasms, Adenomas and Adenocarcinomas, Cystic, Mucinous and Serous Neoplasms)	ADPGK-AS1, AC103591.3, AC018529.1, AC010175.1, AC016747.2, AC007613.1, AC026355.2, ABALON, AC034102.8, AC073316.3, AL031667.3, AC005884.1, TSPOAP1-AS1	(64)
Lung Adenocarcinoma (TCGA-LUAD) (Acinar Cell Neoplasms, Adenomas and Adenocarcinomas, Cystic, Mucinous and Serous Neoplasms)	TMPO-AS1, OGFRP1, LINC01117, HIF1A-AS1, LINC00592, WWC2-AS2, TARID, LINC00628, ABCA9-AS1	(65)
Ovarian cancer (TCGA-OV) (Cystic, Mucinous and Serous Neoplasms)	WACAS1, TRAM2-AS1, SH3RF3-AS1, PCOLCE-AS1, MYCNOS, LINC01270, LINC00592, LAMTOR5-AS1, FOXN3-AS1, DLGAP1-AS2, DICER1-AS1, ARHGAP26-AS1	(66)
Ovarian cancer (TCGA-OV) (Cystic, Mucinous and Serous Neoplasms)	AC008669.1, AC010336.1, AC097376.3, AC130710.1, ACAP2-IT1, AL138820.1, CACNA1G-AS1	(67)
Ovarian cancer (TCGA-OV) (Cystic, Mucinous and Serous Neoplasms)	DNM3OS,WAC-AS1, FOXNS-AS1, LINC00997	(28)
Bladder (TCGA-BLCA) (Adenomas and Adenocarcinomas, Epithelial Neoplasms, NOS, Squamous Cell Neoplasms,Transitional Cell Papillomas and Carcinomas)	PTOV1-AS2, AC116914.2, EHMT2-AS1, AC004148.1, AL136295.2,KCNQ1OT1, AC104564.3, AC073534.2, ATP1B3-AS1	(68)
Bladder (TCGA-BLCA) (Adenomas and Adenocarcinomas, Epithelial Neoplasms, NOS, Squamous Cell Neoplasms,Transitional Cell Papillomas and Carcinomas)	AC006160.1, AC004076.2, BDNF-AS, AC073575.4, AC097641.2, MAP3K14-AS1, ZNF32-AS2, AL136295.2, ZFN436-AS1, AC025280.1, SNHG16, ATP1B3-AS1, AP001469.1, AC005479.1	(69)
Acute myeloid Leukemia (TCGA-LAML)	FAM30A, HCP5, LINC00963,TMEM147-AS1,TTTY15, LINC00342, MEG3, HCG18, N4BP2L2-IT2	(70)
Hepatocellular carcinoma (TCGA-LIHC) (Adenomas and Adenocarcinomas)	ZEB1-AS1, MIR210HG, BACE1-AS, SNHG3	(71)
Hepatocellular carcinoma (TCGA-LIHC) (Adenomas and Adenocarcinomas)	AP001469.3, AL031985.3, SREBF2-AS1, AL442125.2,MKLN1-AS, AL590705.3, TMCC1-AS1, NRAV, C2orf27A, POLH-AS1, AL158166.1, LINC01138, WAC-AS1, AL117336.2	(72)
Colon adenocarcinoma (TCGA-COAD) (Adenomas and Adenocarcinomas, Complex Epithelial Neoplasms, Cystic, Mucinous and Serous Neoplasms, Epithelial Neoplasms, NOS)	AC027307.2, MIR200CHG, RHOA-IT1, AC009996.1, AL138831.2, AC010168.2, AC007066.2, AC019118.1, ALMS1-IT1, UBA6-AS1, SNHG16, FENDRR, RAMP2-AS1, AC013652.1	(73)

Here we review some of the most up to date correlations of m6A-related lncRNAs signatures with survival, progression, and drug resistance of frequently diagnosed human cancers. Recently, by performing coexpression analysis from TCGA and stratifying breast cancer patients into different subgroups, Zhang et al. systematically explored the prognostic and immunotherapeutic value of mRLs. Through several bioinformatic and statistical analyses, they demonstrated the prognostic-immunotherapeutic robustness of a novel signature of twenty-one lncRNAs building up the mRLs model, serving as independent risk factor (56). More detailed studies of Lv et al. and collaborators performed on TCGA database, showed twelve differentially expressed m6A regulator genes in BRCA tissue (compared to normal tissues), and their associated mRLs. Multivariate COX regression analyses showed that all the six lncRNAs examined, were independent prognostic factors for BRCA (four as protective, two as risk factors). Based on risk score, they subsequently divided BRCA patients into the low-risk group and high-risk group and found out that the six mRLs were differentially expressed in these two groups, predicting the 3-year OS (57). Referring to osteosarcoma, Zheng et al., constructed a risk signature, investigating the prognostic independence of the six identified mRLs. The signature was systematically associated with tumor immune microenvironment and immune-cell infiltration. Only one named *AC004812.2* was a protective factor and its lower expression significantly correlated with worse OS (58). For these reasons, they functionally validated this lncRNA in osteosarcoma cell line, showing that its overexpression led to the inhibition of cell proliferation and to the increase in m6A regulators expression (*IGF2BP1, YTHDF1*). Little is known also about mRLs signatures in glioma and gastric cancer, even if recent studies highlighted that abnormal expression of m6A-related genes is associated with their progression. In both cases, recent research highlight promising epigenetic signatures for prognostic purposes (59, 60). Through several bioinformatic and statistical analysis, Wu et al. and collaborators constructed and verified a nine mRLs risk stratification signature for pancreatic ductal adenocarcinoma (PDAC) patients and confirmed its prognostic discriminatory power, highlighting excellent accuracy in predicting 1- and 3-year OS. In this work, they focused on the functional validation of *DCST1-AS1*, already described as EMT-driver in triple-negative breast cancer (74). Its silencing leads to the inhibition of cell proliferation and migration of PDAC cells thus suggesting its crucial role in sustaining PDAC progression (61). Moreover, they showed that this mRLs signature significantly correlates with immune-cell infiltration and sensitivity to chemoterapeutic drugs, adding a new layer of information also for the prediction of therapy response in PDAC patients. Recent evidence suggests that mRLs play a key role also in clear-cell renal cell carcinoma (ccRCC) progression and in dictating immunotherapy efficacy. Referring to ccRCC, Zeng et al. Chen and co-workers previously reported novel prognostic signature based on six lncRNAs and on m6A RNA methylation regulator which display a good prediction power (75, 76). Consistently with these results, Ma et al. and collaborators established a twelve-mRLs prognostic signature for stratifying ccRCC patients, and demonstrated the remarkable prognostic significance of these twelve lncRNAs indicating that were accurate in predicting 1-,3-,5-years OS (62). Recently, Weng et al. identified a mRLs signature for predicting lung squamous cell carcinoma (LUSC) patients’ prognosis, constructing a risk model encompassing only two immune-associated lncRNAs. *AL122125.1* emerged as an independent prognostic factor, providing novel information about LUSC risk-stratification (63). Similar approach has also been applied for the analysis of bladder, colon cancers, hepatocellular carcinoma and hematological malignancies, in particular like acute myelocytic leukemia (28, 64–73, 77, 78).

## 5 m5C-related lncRNAs signatures

Also in the case of m5C the epigenetic signatures of m5C-related lncRNAs show to have prognostic significance (79). The experimental approach is similar to the ones reported for the mRLs signature (**Table 2**).

**Table 2:** m5C-related lncRNAs signatures.

Malignancies	m5C-related lncRNA signature	References
Pancreatic Ductal Adenocarcinoma (TCGA-PDAC)	AC022098.1, AL031775.1, AC005332.6, AC096733.3, AC025165.1, AC009974.1, CASC8 and PAN3-AS1	(34)
Pancreatic Cancer (TCGA-PDAC) (Adenomas and Adenocarcinomas Cystic, Mucinous and Serous Neoplasms Ductal and Lobular Neoplasms, NOS)	TRPC7-AS1, TRAF3IP2-AS1 and AC009974.1	(80)
Lung Adenocarcinoma (TCGA-LUAD)	AC005911.1,AC090948.1, AC106047.1, AC124045.1, AL513550.1, HLA-DQB1-AS1, LINC00654, AL035701.1, LINC00578, SH3BP5-AS1, ABALON, AL034397.3, NKILA, and TMPO-AS1	(81)
Lung Adenocarcinoma (TCGA-LUAD)	LINC00628, LINC02147, and MIR34AHG	(82)
Lung Squamous Cell Carcinoma (TCGA-LUSC)	ERICD, AL021068.1, LINC01341, AC254562.3, and AP002360.1	(83)
Breast Cancer (TCGA-BRCA) (Adenomas and Adenocarcinomas, Adnexal and Skin Appendage Neoplasms, Basal Cell Neoplasms, Complex Epithelial Neoplasms, Ductal and Lobular Neoplasms, Epithelial Neoplasms, NOS, Fibroepithelial Neoplasms, Squamous Cell Neoplasms)	AP005131.2, AL121832.2 and LINC01152	(84)
Prostate Cancer (TCGA-PRAD)	MAFG-AS1, AC012510.1, AC012065.3, AL117332.1, AC132192.2, AP001160.2, AC129510.1, AC084018.2, UBXN10-AS1, AC138956.2, ZNF32-AS2, AC017100.1, AC004943.2, SP2-AS1, Z93930.2, AP001486.2, and LINC01135	(85)
Lower-Grade Gliomas (TCGA-LGG)	LINC00265, CIRBP-AS1, GDNF-AS1, and ZBTB20-AS4	(86)
Lower-Grade Gliomas (LGG)	PAXIP1-AS2, RP11-303E16.2, RP11-157J24.2, RP11-108L7.15, C091878.1, LNC00632, RP11-158M2.3, and CTD-2377O17.1	(87)
Bladder Urothelial Carcinoma (TCGA-BLCA) (Adenomas and Adenocarcinomas, Epithelial Neoplasms, NOS, Squamous Cell Neoplasms, Transitional Cell Papillomas and Carcinomas)	AC004803.1, AC004839.2, AC005229.3, AC007319.1, AC011503.2, AC022001.3, AC024451.4, AC079160.1, AC109449.1, AC124016.1, AL133297.1, AL445228.2, AP003059.1, C2-AS1, HDAC4-AS1, LINC01018 and PCAT7	(88)
Uterine Corpus Endometrial Carcinoma (TCGA-UCEC)	CDKN2B-AS1, NBAT1, NRAV, HM13-IT1, AL078644.2,TTLL11-IT1, FMR1-IT1, NNT-AS1, EMSLR, AC092953.2, YEATS2-AS1, LINC02474, AP001347.1, RAB11B-AS1, BX322234.1, CERNA1 and AL645568.1	(89)
Hepatocellular Carcinoma (TCGA-LIHC)	AC026412.3, AC010969.2, AP003392.5 and SNHG4	(90)
Stomach Adenocarcinoma (TCGA-STAD) (Adenomas and Adenocarcinomas, Cystic, Mucinous and Serous Neoplasms)	AC005586.1, AL590666.2, AP001271.1, IPO5P1, HAGLR and AC009948.1	(91)

Some relevant and recent data are reviewed hereafter. Yuan H. and co-authors demonstrate that, in PDAC, the risk score depends on the m5C-related lncRNAs expression and can predict PDAC patients’ OS. In addition, to determine the risk score, the authors explore the relationship with the immune microenvironment through bioinformatics analysis. Pancreatic cancer and normal tissues also display a significantly different expression of m5C-related lncRNAs. Eight m5C-related lncRNAs and their clinical nomogram predicts 3-years survival time, even though an external database and *in vivo* validations should be performed (34). About two months later, another group of scientists analysed m5C-related lncRNA signature in PDAC. Using TCGA information and bioinformatic analysis, they extracted three m5C-related lncRNAs eventually building a risk signature. One of the three selected lncRNAs (*AC009974.1*) is part of an epithelial-mesenchymal transition-lncRNA signature that predicts the prognosis in PDAC patients. The prognostic nomogram provides also promising immunotherapeutic strategies. Additionally, the authors showed that the patients with high m5C-related lncRNA signature manifest worse prognosis (80). Pan J. and colleagues identified in LUAD 14 m5C-related lncRNAs with prognostic value. Through bioinformatic analysis, they discovered how these 14 m5C-related lncRNAs are immune-related. Moreover, these lncRNAs predict patient prognosis and OS independently of molecular characteristics and clinical risk factors. As they themselves point out, their study presents limitations such as the limited number of datasets, the fact that they validated the prognostic value of lncRNA only at cytological level and the lack of some experiments to confirm m5C modification sites (81). In another article published in 2022, the authors apply the bioinformatic analysis of lung TCGA datasets demonstrating how the m5C score of the lncRNAs is involved in cytoplasmic translation, lymphocyte and endolysosome migration. They detected 16 m5C-related lncRNAs with prognostic relevance. Using LASSO regression, they constructed a prognostic signature consisting of *LINC00628*, *LINC02147*, and *MIR34AHG*. Subsequently, focusing on *LINC00628* they discovered that it correlates with lung progression, since the knockdown of this lncRNA causes a reduction of migration and invasion rates. The limitations of their studies are that they performed only bioinformatics analysis without validation on cross-cohort samples (82). Also in hepatocellular carcinoma, uterine corpus endometrial carcinoma, bladder, breast and prostate cancers and lower-grade gliomas the scientists analysed a specific signature on m5C-lncRNAs. In almost all tumors studied, the signature correlates with cancer metabolism, immune microenvironment and tumor immune-cell infiltration. In some cases, also, with the tumor copy number variation and mutations. The scientists discovered that high m5C score correlates with activation of tumor malignancy-related pathways and decreasing function of immune microenvironment. Overall, the relevant emerging information is that the m5C-related lncRNA signature predicts the patients’ OS. Future directions and clinical studies will unveil whether these lncRNAs could be used as biomarkers and therapeutic targets (83–91). Moreover, the signature is supported by a previous 2020’s pan-cancer study showing that lncRNAs could work as biomarkers including breast, pancreatic, lung and hepatocellular carcinoma (92).

Notably, the lncRNAs could be released in saliva, urine, blood and serum or other body fluids (93). Hence, they feature the potential to be stable biomarkers for tumor prognosis.

A great resource is represented by the recent construction of the m5C-Atlas. The m5C-Atlas currently lacks of the datasets concerning the lncRNAs and the integration with these data will represent a significant improvement to the translatability of these information (94).

## 6 Concluding remarks

Many authors in the last few years focused on the importance of understanding the potential link of m6A and m5C epigenetic modification and lncRNAs in solid and hematological malignancies. Since, the discovery of modified lncRNAs in cancer is still at its infancy, many other investigations are needed to confirm the appropriateness and applicability of these predictive models in other independent patients cohorts. All signatures might provide predictive and/or prognostic tools aimed at improving the molecular knowledge of cancer responsiveness and progression.

## Author contributions

VC: manuscript writing; preparation of figures; AT: manuscript writing; AN: manuscript revision and critical improvement; RF: conceptualization; manuscript writing and revision. All authors contributed to the article and approved the submitted version.
